# Burden of laryngeal cancer attributable to occupational asbestos exposure in China: A comprehensive analysis from 1990 to 2021

**DOI:** 10.1371/journal.pone.0330878

**Published:** 2025-08-21

**Authors:** Bijuan Chen, Zhouwei Zhan, Sisi Yu, Jiali Huang, Chuying Chen, Jie Wang, Jianji Pan, Shaojun Lin, Yun Xu

**Affiliations:** 1 Department of Radiation Oncology, Clinical Oncology School of Fujian Medical University, Fujian Cancer Hospital, Fuzhou, Fujian, China; 2 Department of Medical Oncology, Clinical Oncology School of Fujian Medical University, Fujian Cancer Hospital, Fuzhou, Fujian, China; University of Vermont College of Medicine: University of Vermont Larner College of Medicine, UNITED STATES OF AMERICA

## Abstract

**Background:**

Laryngeal cancer attributable to occupational asbestos exposure remains a significant public health concern, particularly in industrialized regions. This study analyzes the burden, trends, and contributing factors of laryngeal cancer due to asbestos exposure in China from 1990 to 2021.

**Methods:**

Data were obtained from the Global Burden of Disease Study (1990–2021). We analyzed age-standardized death rates, disability-adjusted life years (DALYs), years lived with disability (YLDs), and years of life lost (YLLs). Temporal trends were assessed using joinpoint and decomposition analyses, and an age-period-cohort (APC) model was applied to examine mortality and DALY trends across different cohorts.

**Results:**

In 2021, there were 234 deaths and 4,430 DALYs due to laryngeal cancer attributable to occupational asbestos exposure, predominantly affecting males. Mortality rates declined from 1990 to 2008, followed by a rise until 2012, and a subsequent decline. YLDs showed a consistent increase over time. APC analysis revealed higher mortality and DALY rates in older age groups and earlier birth cohorts. Decomposition analysis indicated that epidemiological changes were the largest driver of increased deaths in men, followed by population growth and aging. For DALYs, aging and population growth were key drivers, while epidemiological changes mitigated the burden.

**Conclusions:**

The burden of laryngeal cancer attributable to asbestos exposure has declined overall, but disability rates continue to rise, particularly among males. Effective strategies targeting prevention, early detection, and management of asbestos exposure are needed to reduce the disease burden in China.

## Introduction

Laryngeal cancer is a serious and often fatal disease, with strong links to occupational exposures, particularly asbestos [1–3]. The International Agency for Research on Cancer (IARC), part of the World Health Organization, has classified asbestos as a Group 1 carcinogen with sufficient evidence in humans for its causal role in laryngeal cancer. Asbestos has been widely used in construction, manufacturing, and other industries due to its durability and fire-resistant properties. However, the inhalation of asbestos fibers has been shown to cause various respiratory diseases, including asbestosis, lung cancer, and mesothelioma [4–6]. More recently, studies have established a connection between asbestos exposure and laryngeal cancer, highlighting the significant occupational risk for individuals working in industries where asbestos use was prevalent [7–9]. Despite global efforts to reduce asbestos usage, its impact continues to be felt in countries like China, where industrial growth has historically been accompanied by widespread asbestos exposure [10,11]. As a result, the incidence of asbestos-related cancers, including laryngeal cancer, remains a pressing public health issue.

Occupational exposure to asbestos is a significant contributor to the burden of laryngeal cancer in China. The country has one of the highest rates of asbestos production and consumption in the world, with millions of workers potentially exposed [12,13]. Previous studies have demonstrated that men, due to their higher likelihood of employment in asbestos-related industries, are disproportionately affected by laryngeal cancer compared to women [14]. Despite regulations aimed at controlling asbestos exposure, the latency period of asbestos-related diseases, often spanning several decades, means that new cases continue to emerge long after exposure has ceased [15]. Additionally, there is a growing body of evidence suggesting that the risk of laryngeal cancer is not limited to direct occupational exposure but may extend to environmental or secondary exposure, further complicating efforts to control the disease [16]. Globally, asbestos remains the leading occupational carcinogen in terms of age-standardized mortality and DALY rates, as highlighted by Zou et al. in their analysis of GBD 2021 data [17]. Their findings underscore the persistent global burden of asbestos-related cancers despite declining trends in high-income countries.

Our study contributes to this landscape by focusing on laryngeal cancer, a less commonly examined but increasingly recognized outcome of occupational asbestos exposure, particularly relevant in middle-SDI countries such as China. Despite ongoing research, there is a lack of comprehensive data on the long-term trends in mortality, disability-adjusted life years (DALYs), years lived with disability (YLDs), and years of life lost (YLLs) attributable to asbestos exposure in China. Previous global studies have focused predominantly on lung cancer and mesothelioma, often neglecting the role of asbestos in laryngeal cancer [18,19]. This study aims to fill this gap by providing a detailed analysis of the burden of laryngeal cancer attributable to occupational asbestos exposure in China from 1990 to 2021. By employing methods such as joinpoint regression and decomposition analysis, this study seeks to highlight key temporal trends and identify the factors driving changes in the disease burden. The results of this research will inform future efforts to reduce asbestos exposure and mitigate the health impacts in affected populations.

## Methods

### Data source

This study is based on data from the Global Burden of Disease (GBD) 2021 Study, developed by the Institute for Health Metrics and Evaluation (IHME), which provides comprehensive, standardized estimates of disease burden across 204 countries and territories from 1990 to 2021 [20,21]. The data are publicly available through the Global Health Data Exchange (GHDx) query tool (https://ghdx.healthdata.org/gbd-results-tool). For this analysis, we extracted data on laryngeal cancer attributable specifically to occupational asbestos exposure in China between 1990 and 2021, including estimates of deaths, DALYs, YLDs, and YLLs, as well as their corresponding age-standardized rates [20]. The GBD employs a comprehensive methodology integrating data from multiple sources, including vital registration systems, cancer registries, population-based surveys, and occupational exposure data to estimate the burden of disease. Cause of death data were modeled using the Cause of Death Ensemble model (CODEm), and non-fatal health outcomes were estimated using DisMod-MR 2.1, a Bayesian meta-regression tool. All estimates are reported with 95% uncertainty intervals (UIs), reflecting uncertainty from data sampling, model selection, and covariate inputs. This dataset allows for a robust and comparable assessment of the long-term burden of laryngeal cancer linked to asbestos exposure, including time trends, sex-specific patterns, and risk-attributable metrics, enabling further epidemiological and policy-relevant evaluations.

### Definition and estimation

In this study, the burden of laryngeal cancer attributable to occupational asbestos exposure was estimated based on the comparative risk assessment framework of the GBD Study 2021. Occupational asbestos exposure was defined using the asbestos impact ratio (AIR), which represents the ratio of excess deaths from mesothelioma observed in a given population to those in a reference population with high asbestos exposure. This proxy enables estimation of population-level asbestos exposure across countries and time. The burden was quantified using four key indicators: deaths, DALYs, YLDs, and YLLs, expressed in both absolute numbers and age-standardized rates per 100,000 population. Population attributable fractions (PAFs) were calculated by integrating exposure estimates with relative risks (RRs) obtained from meta-analyses of epidemiological studies. Attributable deaths and DALYs were then estimated by multiplying the total number of laryngeal cancer cases by the corresponding PAFs. DALYs were derived as the sum of YLDs and YLLs, with YLDs calculated by multiplying the prevalence of laryngeal cancer by a disability weight specific to the condition, which quantifies the severity of health loss on a scale from 0 (full health) to 1 (equivalent to death). YLLs were computed by multiplying the number of deaths at each age by the standard life expectancy at that age, using a GBD reference life table based on the lowest observed mortality rates globally. This standard assumes a life expectancy at birth of 86.0 years for males and 88.9 years for females [22]. All estimates included 95% UIs, generated using 1,000 posterior draws to reflect uncertainty in input data, model parameters, and exposure – risk relationships.

### Descriptive analysis

We conducted a descriptive analysis to assess the burden of laryngeal cancer attributable to occupational asbestos exposure in China from 1990 to 2021. The primary indicators included the number and age-standardized rates (per 100,000 population) of deaths, DALYs, YLDs, and YLLs, stratified by sex and age group. Data were visualized using line graphs and age-specific plots to illustrate temporal trends and demographic patterns. Figures were constructed to compare absolute numbers versus standardized rates to account for the effects of population growth and aging. In addition, age-specific distributions for the year 2021 were examined to identify the age groups with the greatest burden. Sex-specific disparities were also explored to highlight the disproportionate impact on males. The results were summarized using measures of central tendency and range, with all estimates accompanied by 95% UIs. This descriptive approach provided the foundation for identifying key trends and burden shifts over time and guided the subsequent application of trend and decomposition analyses.

### Joinpoint regression analysis

Joinpoint regression analysis was employed to assess temporal trends in age-standardized rates of deaths, DALYs, YLDs, and YLLs due to laryngeal cancer attributable to occupational asbestos exposure in China from 1990 to 2021. This method identifies points in time, known as “joinpoints”, where statistically significant changes in trend occur and quantifies the magnitude of these changes using the annual percentage change and average annual percentage change (AAPC). We used the Joinpoint Regression Program (version 5.2.0) developed by the US National Cancer Institute, applying a log-linear model with a maximum of three joinpoints permitted. Statistical significance was determined using a Monte Carlo permutation method with a significance threshold set at *p* < 0.05 [23,24]. This approach enabled us to detect shifts in the trend patterns and evaluate whether the observed changes over time reflected acceleration, deceleration, or stabilization in the burden. Analyses were conducted separately for males and females to capture sex-specific dynamics in the burden evolution.

### Age-period-cohort (APC) analysis

To disentangle the independent effects of age, time period, and birth cohort on the trends in laryngeal cancer burden attributable to occupational asbestos exposure, we performed APC analysis using mortality and DALY data from 1990 to 2021. Mortality and DALY data were stratified into consecutive 5-year age groups (from 20–24 to 90–94 years), 5-year time periods (from 1992–1996 to 2017–2021), and corresponding 5-year birth cohorts. The intrinsic estimator (IE) method was used to address the collinearity problem among age, period, and cohort variables. This method provides unbiased and interpretable estimates of age, period, and cohort effects in log-linear models [25]. The analysis was conducted separately for deaths and DALYs and stratified by sex to explore gender-specific temporal patterns. We used the APC tools implemented in R software (version 4.3.1), applying Poisson regression models with log link functions. Results were visualized to demonstrate trends in age-specific rates across periods and cohorts, as well as cohort-specific rates across age groups. This approach enabled us to evaluate how demographic and temporal factors contributed to the observed changes in disease burden, particularly the increased burden among older age groups and earlier birth cohorts.

### Decomposition analysis

To quantify the drivers behind the changes in the burden of laryngeal cancer attributable to occupational asbestos exposure in China between 1990 and 2021, we conducted a decomposition analysis. This method breaks down the net change in the number of deaths and DALYs into contributions from three components: population growth, population aging, and epidemiological changes. In this context, epidemiological changes refer to variations in disease risk due to factors such as improvements in occupational health regulations, early detection, treatment advances, and changes in the distribution or intensity of asbestos exposure. The decomposition was performed using a standard decomposition algorithm adapted from previous GBD-related analyses [26,27]. The total change in absolute burden over time was sequentially attributed to changes in population size, shifts in age structure, and age-specific rates of burden. The effect of population growth was assessed by holding the age structure and rates constant, while the aging effect was isolated by holding rates constant and adjusting for changes in age composition. The residual was attributed to epidemiological changes. The analysis was conducted separately for males and females, allowing for comparison of the relative influence of each factor by sex. Visualization of the results was used to highlight dominant contributors to the increase or decrease in burden over the three-decade period.

### Ethical considerations

The data used in this study were publicly available and did not require ethical approval. All analyses followed the GBD guidelines for accurate and transparent health assessment reporting.

## Results

### Burden of laryngeal cancer attributable to occupational asbestos exposure in China, 2021

In 2021, laryngeal cancer attributable to occupational asbestos exposure in China showed a pronounced gender disparity, with males experiencing the bulk of the burden. There were 234 total deaths, 195 of which were males, compared to 39 in females (Table 1). The age-standardized death rate for males was 0.02 per 100,000, while the rate for females was close to zero. DALYs amounted to 4,430, with males contributing 3,725 and females 705. The age-standardized DALY rate was 0.4 per 100,000 for males and 0.06 for females. YLDs were relatively low, with a rate of 0.02 per 100,000 for males, indicating minimal long-term disability, and a negligible rate for females. The greatest burden was seen in YLLs, with a total of 4,239, emphasizing the premature mortality associated with asbestos-related laryngeal cancer, particularly in males (Table 1).

**Table 1:** All-age cases and age-standardized deaths, DALYs, YLDs, and YLLs rates in 2021 for larynx cancer attributable to occupational asbestos exposure in China.

Measure	All-ages cases			Age-standardized rates per 100,000 people		
	Total	Male	Female	Total	Male	Female
Deaths	234 (123, 379)	195 (90, 334)	39 (12, 74)	0.01 (0.01, 0.02)	0.02 (0.01, 0.04)	0 (0, 0.01)
DALYs	4430 (2267, 7341)	3725 (1671, 6584)	705 (215, 1357)	0.21 (0.11, 0.35)	0.4 (0.18, 0.69)	0.06 (0.02, 0.12)
YLDs	191 (100, 336)	156 (69, 285)	35 (10, 68)	0.01 (0, 0.02)	0.02 (0.01, 0.03)	0 (0, 0.01)
YLLs	4239 (2158, 7025)	3569 (1600, 6303)	670 (205, 1283)	0.2 (0.1, 0.33)	0.39 (0.18, 0.66)	0.06 (0.02, 0.12)

Values in parentheses indicate 95% UIs, estimated using Monte Carlo simulations. Abbreviations: DALYs, disability-adjusted life-years; YLDs, years lived with disability; YLLs, years of life lost; UIs, uncertainty intervals.

**Fig 1:**
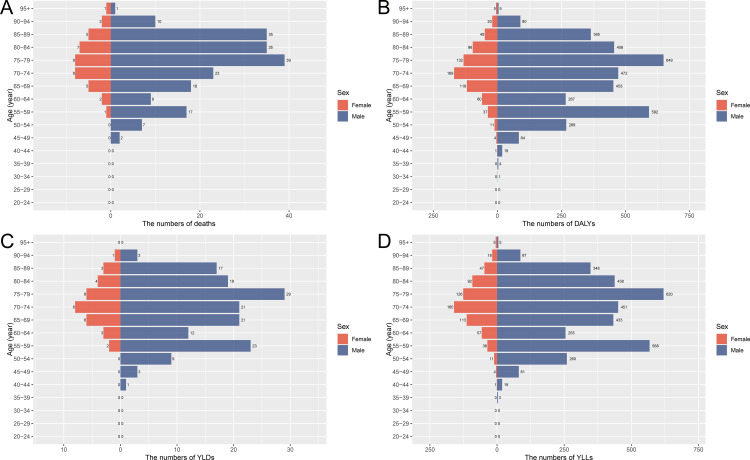
Age-specific numbers of deaths, DALYs, YLDs, and YLLs due to laryngeal cancer attributable to occupational asbestos exposure in China, 2021, by sex. **(A)** Number of deaths. **(B)** Number of DALYs. **(C)** Number of YLDs. **(D)** Number of YLLs. Abbreviations: DALYs, disability-adjusted life years; YLDs, years lived with disability; YLLs, years of life lost.

**Fig 2:**
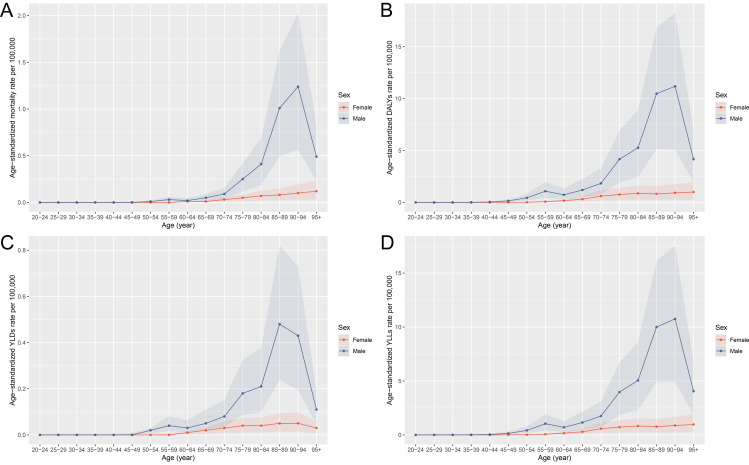
Age-specific rates of deaths, DALYs, YLDs, and YLLs due to laryngeal cancer attributable to occupational asbestos exposure in China, 2021, by sex. **(A)** Rate of deaths. **(B)** Rate of DALYs. **(C)** Rate of YLDs. **(D)** Rate of YLLs. Abbreviations: DALYs, disability-adjusted life years; YLDs, years lived with disability; YLLs, years of life lost.

The data in Figs 1-2 illustrate that the burden of laryngeal cancer, in terms of deaths, DALYs, YLDs, and YLLs, is primarily concentrated in individuals aged 65–89, with a notable peak observed in the 55–59 age group. Fig 1 shows that deaths and DALYs sharply increase in males within these age groups, while females exhibit much lower and more stable patterns. YLLs follow a similar trend, peaking significantly in males aged 55–59 and 65–69, reflecting the considerable impact of premature mortality (Fig 1). In terms of age-specific rates (Fig 2), males demonstrate a steep rise in all four indicators after age 50, with the highest rates occurring between ages 65–89, and a secondary peak in the 55–59 age group. Females show much lower rates across all age groups, reinforcing the disproportionate burden among males. In summary, occupational asbestos exposure contributes significantly to the burden of laryngeal cancer in China, with the most substantial impact seen in males aged 55–89. The indicators of disease burden are concentrated in this older population, particularly reflecting the heavy toll of premature mortality and YLLs.

### Temporal trends in the burden of laryngeal cancer attributable to occupational asbestos exposure from 1990 to 2021

**Fig 3:**
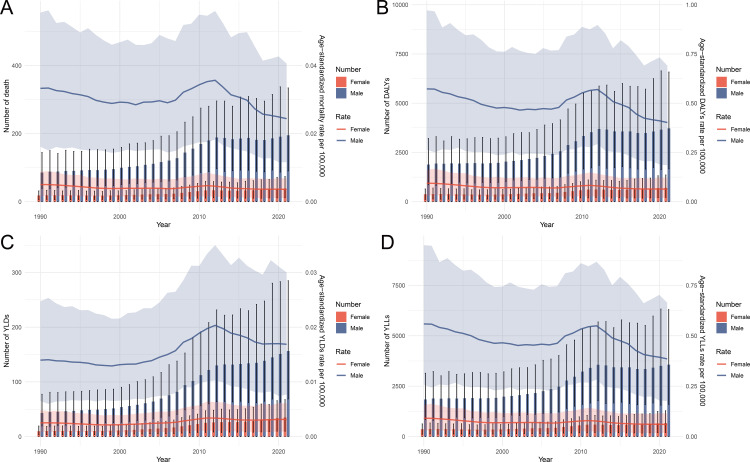
Temporal trends in deaths, DALYs, YLDs, and YLLs due to laryngeal cancer attributable to occupational asbestos exposure in China, 1990-2021, by sex. **(A)** Number of deaths and age-standardized death rates per 100,000 population. **(B)** Number of DALYs and age-standardized DALY rates per 100,000. **(C)** Number of YLDs and age-standardized YLD rates per 100,000. **(D)** Number of YLLs and age-standardized YLL rates per 100,000. Abbreviations: DALYs, disability-adjusted life years; YLDs, years lived with disability; YLLs, years of life lost.

From 1990 to 2021, the burden of laryngeal cancer attributable to occupational asbestos exposure in China exhibited a complex temporal trend. The overall burden showed a downward trend from 1990 to 2008, followed by a rise in 2008 that peaked in 2012, after which it gradually declined. The number of deaths and age-standardized death rates saw a steady decrease until 2008, after which they increased sharply, peaking in 2012, especially among males, before declining in subsequent years (Fig 3A). DALYs followed a similar pattern, with a downward trend until 2008, then a significant rise, peaking in 2012, and a subsequent decline, with males bearing the largest burden (Fig 3B). YLDs showed a modest increase, peaking in 2012 and slightly declining afterward, with males consistently showing higher rates than females (Fig 3C). Finally, YLLs displayed the same overall pattern, with a steady decrease until 2008, a sharp rise to a peak in 2012, followed by a gradual decline, particularly in males (Fig 3D). These trends suggest a temporary increase in the burden of asbestos-related laryngeal cancer post-2008, which peaked in 2012, followed by a gradual reduction in the burden.

### Comparison of age-specific burden of laryngeal cancer attributable to occupational asbestos exposure between 1990 and 2021

**Fig 4:**
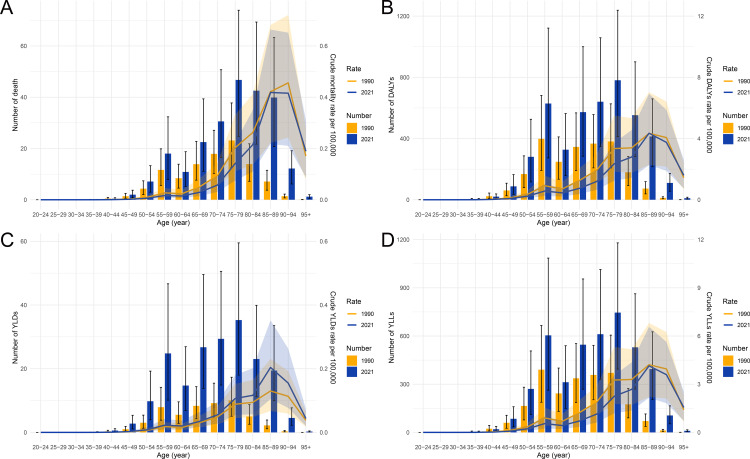
Age-specific numbers and crude rates of deaths, DALYs, YLDs, and YLLs due to laryngeal cancer attributable to occupational asbestos exposure in China, in 1990 and 2021. **(A)** Number of deaths and crude death rates per 100,000 population. **(B)** Number of DALYs and crude DALY rates per 100,000. **(C)** Number of YLDs and crude YLD rates per 100,000. **(D)** Number of YLLs and crude YLL rates per 100,000. Abbreviations: DALYs, disability-adjusted life years; YLDs, years lived with disability; YLLs, years of life lost.

In the comparison of the burden of laryngeal cancer attributable to occupational asbestos exposure between 1990 and 2021, most indicators showed a decline, except for YLDs, which exhibited an upward trend. The number of deaths and crude death rates per 100,000 population generally decreased across age groups in 2021 compared to 1990, with the largest impact seen in the 60–79 age range (Fig 4A). A similar decline was observed in DALYs and their rates, with a significant reduction in 2021, particularly in individuals aged 60–79 (Fig 4B). In contrast, YLDs displayed a rise in both number and crude rates, with the most substantial increase occurring in the 60–74 age group, reflecting a growing burden of long-term disability despite improvements in mortality (Fig 4C). The YLLs showed a noticeable decline in 2021, particularly in the older population (75–89 years), emphasizing a reduction in premature mortality compared to 1990 (Fig 4D). Overall, the data suggest improvements in mortality and life-years lost but highlight an increasing trend in disability burden over time.

### Comparison of global and Chinese trends in age-standardized rates of laryngeal cancer attributable to occupational asbestos exposure

From 1990 to 2021, both global and China-specific trends in laryngeal cancer attributable to occupational asbestos exposure exhibited a general decline in most indicators, with the exception of YLDs, which showed a slight increase in China. As outlined in Table 2, the age-standardized death rate in China decreased from 0.02 in 1990 to 0.01 in 2021, while globally, the decrease was more substantial, from 0.08 to 0.04. DALYs in China fell from 0.3 to 0.21 per 100,000, while globally they dropped more steeply, from 1.58 to 0.78. YLDs in China increased slightly from 0.01 in 1990 to 0.01 in 2021, reflecting a growing disability burden, while global YLDs declined. The YLLs also decreased in both China and globally, with China’s rate falling from 0.29 to 0.2 and the global rate dropping from 1.52 to 0.73 per 100,000. S1 Fig further illustrates these trends, showing a clear downward trajectory in mortality, DALYs, and YLLs in both China and globally, while YLDs in China showed a slight upward trend. This comparison highlights the overall reduction in mortality and YLLs globally and in China, with a rising disability burden particularly noted in China.

**Table 2:** Change of age-standardized rates in deaths, DALYs, YLDs, and YLLs for larynx cancer attributable to occupational asbestos exposure between 1990 and 2021 in China and global level.

Measure	China			Global		
	1990	2021	Change	1990	2021	Change
Deaths	0.02 (0.01, 0.03)	0.01 (0.01, 0.02)	−0.99 (−1.29 - −0.70) ^*^	0.08 (0.04, 0.11)	0.04 (0.02, 0.06)	−2.03 (−2.21 - −1.85) ^*^
DALYs	0.3 (0.16, 0.48)	0.21 (0.11, 0.35)	−1.17 (−1.41 - −0.92) ^*^	1.58 (0.91, 2.37)	0.78 (0.43, 1.18)	−2.28 (−2.40 - −2.16) ^*^
YLDs	0.01 (0, 0.01)	0.01 (0, 0.02)	0.68 (0.41 - 0.94) ^*^	0.07 (0.04, 0.1)	0.04 (0.02, 0.07)	−1.43 (−1.55 - −1.31) ^*^
YLLs	0.29 (0.16, 0.47)	0.2 (0.10, 0.33)	−1.23 (−1.47 - −0.98) ^*^	1.52 (0.87, 2.28)	0.73 (0.41, 1.12)	−2.32 (−2.45 - −2.20) ^*^

Values in parentheses indicate 95% UIs, estimated using Monte Carlo simulations. Abbreviations: DALYs, disability-adjusted life-years; YLDs, years lived with disability; YLLs, years of life lost; UIs, uncertainty intervals; ^*^, **p* *< 0.05.

### Trends in age-standardized rates of laryngeal cancer attributable to occupational asbestos exposure in China based on Joinpoint analysis

**Fig 5:**
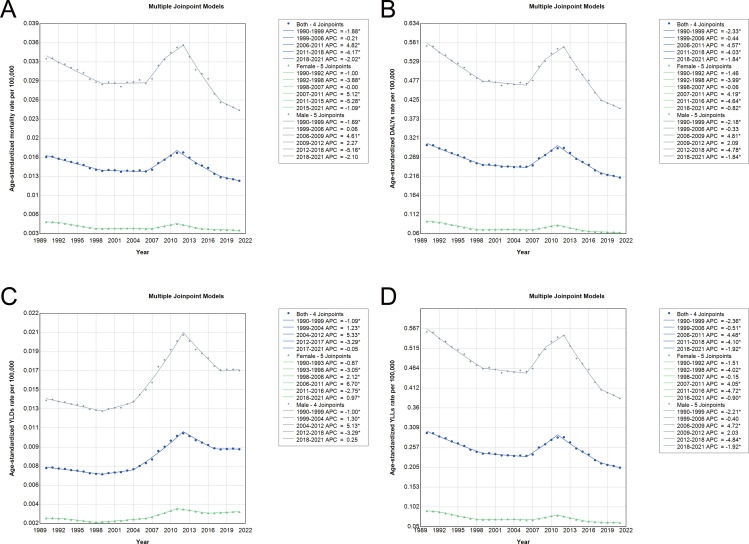
Joinpoint analysis of trends in age-standardized rates of laryngeal cancer attributable to occupational asbestos exposure in China, 1990-2021. **(A)** Age-standardized mortality rate. **(B)** Age-standardized DALYs rate. **(C)** Age-standardized YLDs rate. **(D)** Age-standardized YLLs rate. Abbreviations: DALYs, disability-adjusted life years; YLDs, years lived with disability; YLLs, years of life lost.

The joinpoint analysis of age-standardized rates for laryngeal cancer attributable to occupational asbestos exposure in China between 1990 and 2021 reveals distinct trends across various indicators. Age-standardized mortality rates demonstrated a consistent decline in the earlier years, with significant shifts occurring from 2006 to 2011, where a temporary increase was observed, followed by a steady decline from 2011 onwards (Fig 5A). A similar pattern was observed for DALYs, where a sharp rise occurred between 2006 and 2011, peaking during this period, followed by a notable decrease, particularly after 2011 (Fig 5B). The YLDs exhibited a more stable upward trend throughout the study period, indicating a continuous rise in the burden of long-term disability (Fig 5C). In contrast, YLLs showed a significant decline after 2011, reflecting improvements in reducing premature mortality (Fig 5D). As detailed in S1 Table, the trends in age-standardized rates were different for males and females. Both sexes experienced a decline in age-standardized mortality and DALYs rates, with females showing a steeper reduction. The rise in YLDs, however, was more pronounced in males, with significant increases between 2006 and 2011. In the case of YLLs, both sexes saw improvements, particularly after 2011, with a notable decrease in premature mortality across all groups. These results suggest a complex trend in the burden of laryngeal cancer, where improvements in mortality and life lost were evident, but the disability burden continued to rise.

### APC analysis of mortality and DALYs for laryngeal cancer attributable to occupational asbestos exposure

**Fig 6:**
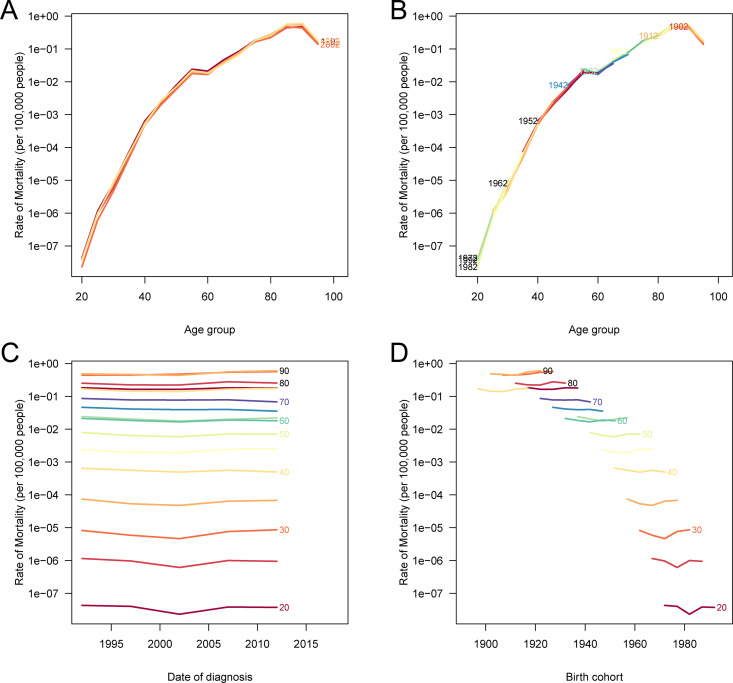
Age-period-cohort analysis of deaths due to laryngeal cancer attributable to occupational asbestos exposure in China. **(A)** Age-specific mortality rates according to time periods; each line connects the age-specific mortality rates for a 5-year period. **(B)** Age-specific mortality rates according to birth cohorts; each line connects the age-specific mortality rates for a 5-year birth cohort. **(C)** Period-specific mortality rates according to age groups; each line connects the period-specific mortality rates for a 5-year age group. **(D)** Birth cohort-specific mortality rates according to age groups; each line connects the birth cohort-specific mortality rates for a 5-year age group.

The APC analysis of mortality and DALYs due to laryngeal cancer attributable to occupational asbestos exposure in China reveals distinct trends across age, period, and cohort effects. As shown in Fig 6, age-specific mortality rates increased steadily with advancing age across all periods, with the highest rates observed in older age groups (Fig 6A). Mortality rates also varied significantly by birth cohort, with earlier cohorts exhibiting higher mortality risks compared to more recent cohorts (Fig 6B). Period-specific mortality rates, when stratified by age group, demonstrated fluctuations across time, with a notable increase in mortality during certain periods (Fig 6C). Similarly, S2 Fig highlights the age-specific DALY rates, showing that older age groups consistently experienced higher DALY rates across time periods (S2A Fig). The cohort-specific analysis of DALYs shows a higher disease burden in earlier birth cohorts, similar to mortality trends (S2B Fig). These results emphasize the substantial burden on older age groups and earlier birth cohorts, underscoring the influence of occupational asbestos exposure on long-term health outcomes.

### Decomposition of changes in deaths and DALYs for laryngeal cancer attributable to occupational asbestos exposure

**Fig 7:**
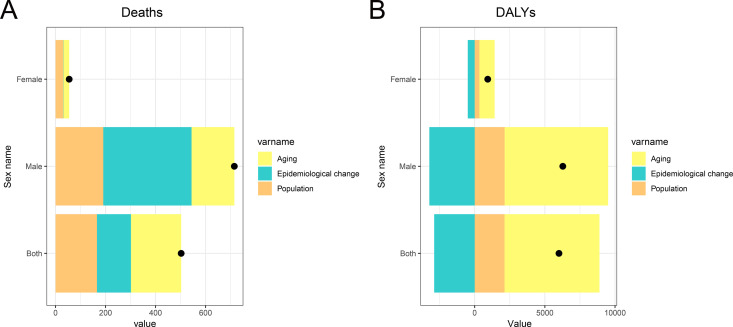
Decomposition analysis of changes in deaths and DALYs due to laryngeal cancer attributable to occupational asbestos exposure in China from 1990 to 2021, by sex. The black dots represent the cumulative effect of these three factors. **(A)** Decomposition of the changes in deaths. **(B)** Decomposition of the changes in DALYs. Abbreviations: DALYs, disability-adjusted life years.

The decomposition analysis of changes in deaths and DALYs due to laryngeal cancer attributable to occupational asbestos exposure in China from 1990 to 2021 reveals that aging, population growth, and epidemiological changes were all key drivers of the changes in mortality metrics, with a significantly greater impact on men than women. As shown in Fig 7A, among men, epidemiological changes had the greatest effect on the increase in deaths, followed by population growth and aging. In contrast, for women, the influence of these factors was less pronounced, but aging still played a significant role. In Fig 7B, for DALYs, epidemiological changes served as a mitigating factor, reducing the overall burden, while both aging and population growth were drivers of increased DALYs. Aging had a greater impact than population growth in both sexes, and all three factors had a more substantial effect on men than on women, reflecting the heavier burden of asbestos exposure among males.

## Discussion

This study provides a comprehensive analysis of the burden and temporal trends of laryngeal cancer attributable to occupational asbestos exposure in China from 1990 to 2021. Our findings demonstrate a significant decline in mortality, DALYs, and YLLs after peaking in 2012, while YLDs, though they have declined since 2012, remain relatively high, indicating a persistent disability burden. The burden is disproportionately higher in males compared to females, reflecting the historical occupational exposure to asbestos in male-dominated industries. The APC analysis revealed that older age groups and earlier birth cohorts experienced higher mortality and DALYs, with epidemiological changes emerging as the primary factor contributing to the rise in deaths among males. Decomposition analysis further underscored the role of aging and population growth as drivers of the increased burden, while improvements in epidemiological factors mitigated the overall impact on DALYs. These findings suggest that while progress has been made in reducing premature mortality, the rising disability burden presents new challenges for public health efforts in asbestos-related disease prevention and management.

Our study contributes to the limited but growing literature on the burden of laryngeal cancer attributable to occupational asbestos exposure in China. Although the absolute number of deaths is relatively low, the burden remains a concern due to its disproportionate impact on older male populations and the persistence of disability-related outcomes. Comparative analysis indicates that age-standardized rates of death and DALYs in China are lower than global averages, likely reflecting recent regulatory efforts and increasing awareness of asbestos-related health risks in the country [12,28,29]. Nonetheless, global indicators have shown a more pronounced decline over time, suggesting that more stringent asbestos control and occupational health practices elsewhere may be yielding stronger results [12]. Although age-standardized rates have declined both in China and worldwide, the disease burden linked to asbestos exposure, especially regarding years lived with disability and among high-risk populations, continues to be a pressing concern and underscores the importance of ongoing public health efforts.

The mechanisms underlying laryngeal cancer caused by occupational asbestos exposure are well-documented. Asbestos fibers, when inhaled, become lodged in the respiratory tract and larynx, causing chronic inflammation, fibrosis, and eventually malignant transformation of epithelial cells [9]. Furthermore, extended exposure to chrysotile (one type of asbestos) may lead to the inactivation of the tumor suppressor genes P53 and P16 as well as the activation of the proto-oncogenes C-JUN and C-FOS at both the messenger RNA and protein levels [30]. This process can take decades to manifest, which explains the long latency period observed in asbestos-related cancers. Preventive measures should focus on minimizing occupational exposure through stricter regulations on asbestos use, better workplace safety practices, and early screening for at-risk populations. Additionally, educating workers about the risks of asbestos exposure and promoting the use of protective equipment in high-risk industries are essential for reducing the incidence of laryngeal cancer [31–33].

A key finding of our study is the marked gender disparity, with males experiencing a significantly greater burden of laryngeal cancer attributable to occupational asbestos exposure. This difference is likely due to the higher prevalence of male employment in asbestos-related industries such as construction, shipbuilding, and manufacturing, where direct exposure is more common [34]. Although women are less commonly employed in high-risk industrial occupations, secondary exposure through environmental proximity to industrial zones or contact with contaminated clothing may help account for the lower, yet still appreciable, burden observed among females [35]. The APC analysis offered important insights into temporal dynamics, revealing a sharp increase in burden between 2006 and 2011. This pattern likely reflects the long latency period of asbestos-related cancers and occupational exposures that occurred during China’s rapid industrial expansion in the late 20th century [36,37]. The observed decline after 2012 may suggest the early benefits of enhanced regulatory measures and improved occupational safety practices. Nevertheless, the continued presence of disease burden in older male cohorts underscores the lasting health impact of historical asbestos exposure and the importance of long-term surveillance and preventive efforts.

Although this study is descriptive in nature and does not assess the effectiveness of specific interventions, the documented burden of laryngeal cancer attributable to asbestos exposure, particularly among older males, underscores the necessity of implementing targeted prevention and control strategies. Enhanced enforcement of existing asbestos regulations through systematic inspections and compliance monitoring could help curtail ongoing exposure risks. Over time, phasing out asbestos use entirely, as adopted by numerous other nations, may yield substantial public health benefits. Routine surveillance of individuals employed in high-risk occupations, using tools such as low-dose computed tomography or laryngoscopy, could facilitate earlier identification of asbestos-related diseases, though the utility of such methods in screening for laryngeal cancer warrants further validation. Maintaining detailed occupational health records may also assist in the longitudinal assessment of cumulative exposure. Furthermore, public education initiatives aimed at increasing awareness of asbestos-related hazards, including indirect and environmental exposure pathways, could foster greater engagement in protective practices. While additional research is needed to evaluate the efficacy of these approaches, they offer promising directions for reducing disease burden among vulnerable populations [15,34].

While this study offers valuable insights into the burden of laryngeal cancer attributable to occupational asbestos exposure in China, several limitations must be considered. First, reliance on GBD 2021 estimates introduces inherent uncertainties, as the accuracy of modeled outputs depends on the quality and availability of input data. However, the GBD framework applies rigorous statistical methodologies, including CODEm and DisMod-MR 2.1, to integrate multiple data sources and adjust for underreporting and data gaps, thereby enhancing internal validity and comparability over time. Second, while underdiagnosis and limited access to healthcare in rural areas may contribute to underreporting of laryngeal cancer cases, the inclusion of uncertainty intervals around each estimate helps account for this variability. Third, this study focuses specifically on occupational asbestos exposure and does not assess other potential risk factors such as smoking, alcohol use, or environmental pollutants. Nonetheless, the GBD’s comparative risk assessment framework enables the estimation of burden attributable to specific exposures while adjusting for coexisting risks at the population level. Finally, although asbestos-related diseases are characterized by long latency periods, the three-decade time frame of our analysis helps accommodate this temporal delay and supports the validity of observed trends. Future research should incorporate individual-level exposure histories, longitudinal cohort designs, and granular data on environmental and behavioral factors to refine risk attribution. Qualitative studies exploring the lived experiences of high-risk workers may also provide valuable insights into prevention, surveillance, and healthcare access. Strengthening public health data infrastructure and fostering interdisciplinary collaboration will be essential to guide evidence-based asbestos control strategies.

## Conclusions

This study underscores the persistent public health challenge posed by laryngeal cancer attributable to occupational asbestos exposure in China. The findings highlight the need for targeted interventions aimed at older male populations with historical occupational exposure, who continue to bear a disproportionate burden of this disease. Future research should focus on expanding the understanding of various risk factors, including environmental and lifestyle influences, to develop comprehensive prevention strategies. Additionally, qualitative studies exploring the lived experiences of affected individuals can provide valuable insights into effective health-seeking behaviors and barriers to care. Enhancing data collection methods and collaboration between public health officials and researchers will be essential for implementing evidence-based policies aimed at reducing the incidence of laryngeal cancer. Ultimately, a multifaceted approach that combines regulation, public health education, and ongoing research is necessary to mitigate the impact of asbestos-related diseases and improve health outcomes in high-risk populations.

## Supporting information

S1 Fig Global and Chinese trends in age-standardized rates of laryngeal cancer attributable to occupational asbestos exposure, 1990–2021. (A) Global trends. (B) Trends in China.
(PDF)

S2 Fig Age-period-cohort analysis of DALYs due to laryngeal cancer attributable to occupational asbestos exposure in China. (A) Age-specific DALY rates according to time periods; each line connects the age-specific DALY rates for a 5-year period. (B) Age-specific DALY rates according to birth cohorts; each line connects the age-specific DALY rates for a 5-year birth cohort. (C) Period-specific DALY rates according to age groups; each line connects the period-specific DALY rates for a 5-year age group. (D) Birth cohort-specific DALY rates according to age groups; each line connects the birth cohort-specific DALY rates for a 5-year age group. Abbreviations: DALYs, disability-adjusted life years.
(PDF)

S1 Table Trends in age-standardized mortality, DALY, YLD, and YLL rates (per 100,000 persons) among both sexes, males, and females from 1990 to 2021 for larynx cancer attributable to occupational asbestos exposure in China. (DOCX)
