# Supplementary figures and images for: Burden of laryngeal cancer attributable to occupational asbestos exposure in China: A comprehensive analysis from 1990 to 2021
Source: PLoS One. 2025 Aug 21;20(8):e0330878. doi: 10.1371/journal.pone.0330878 (PMC12370065; doi:10.1371/journal.pone.0330878)

**A** 1990–2021 Global Age-standardized Rate

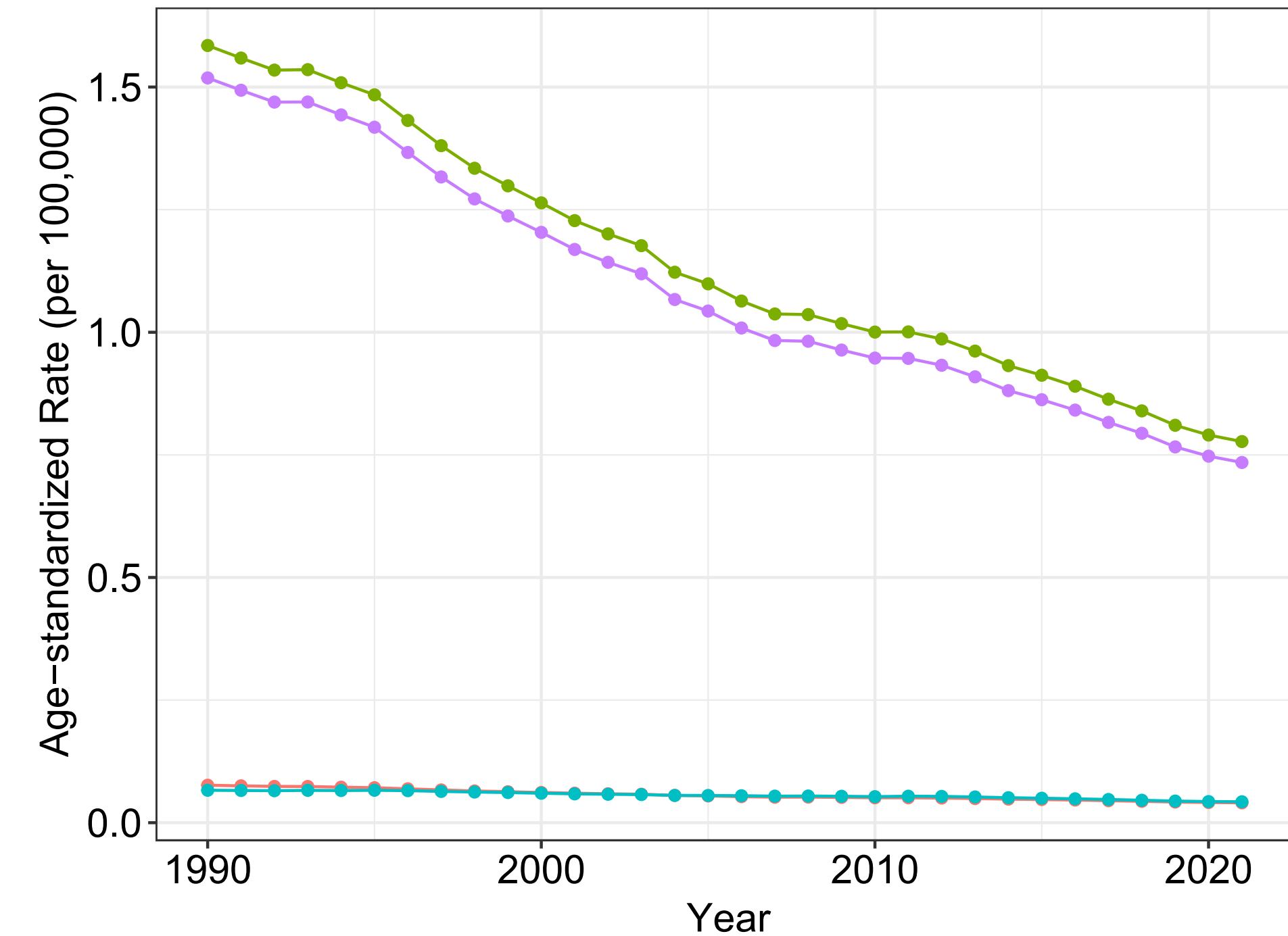

**B** 1990–2021 China Age-standardized Rate

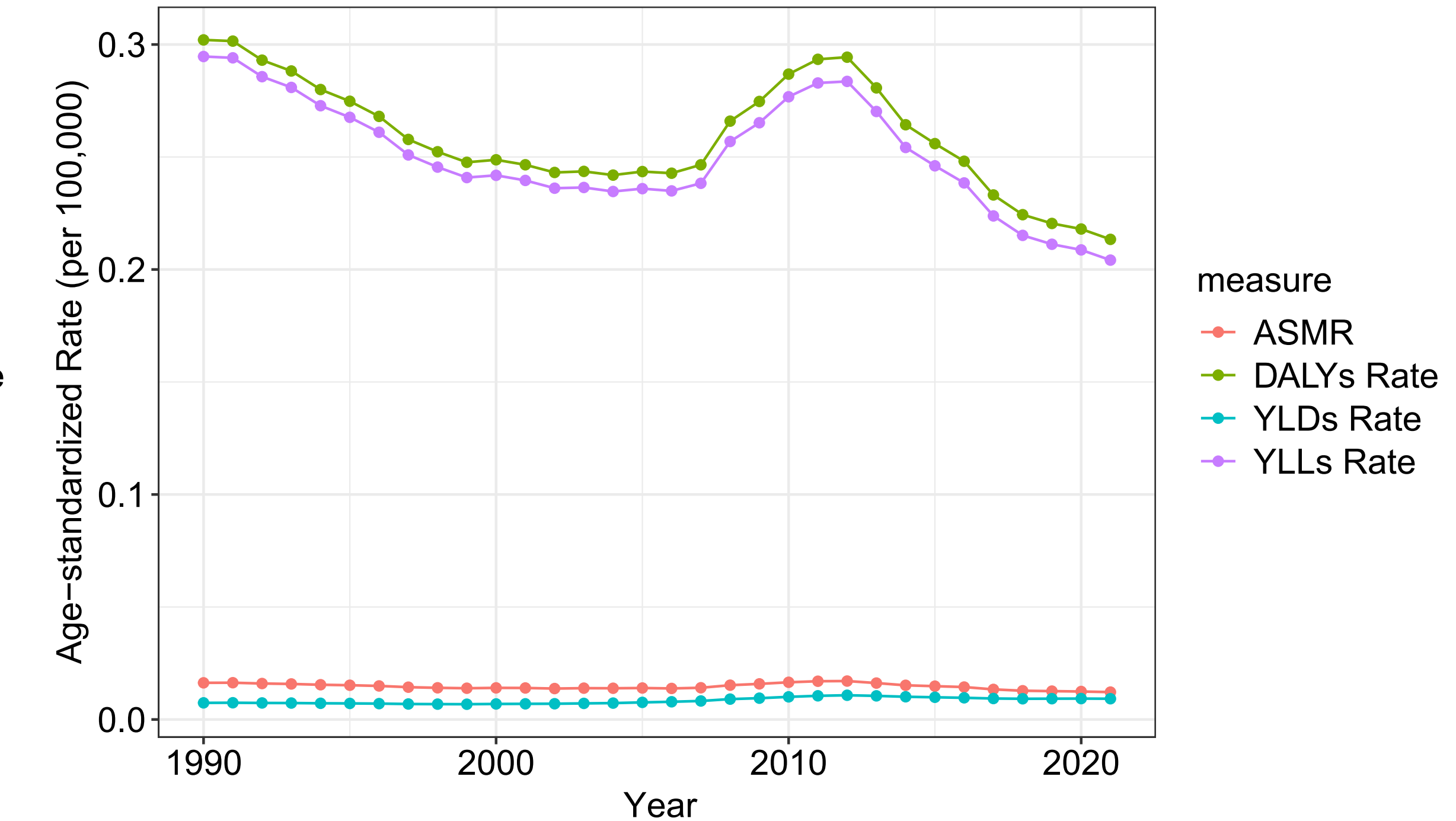

Supplement: S1 Fig — (A) Global trends. (B) Trends in China. (PDF) [file pone.0330878.s001.pdf]

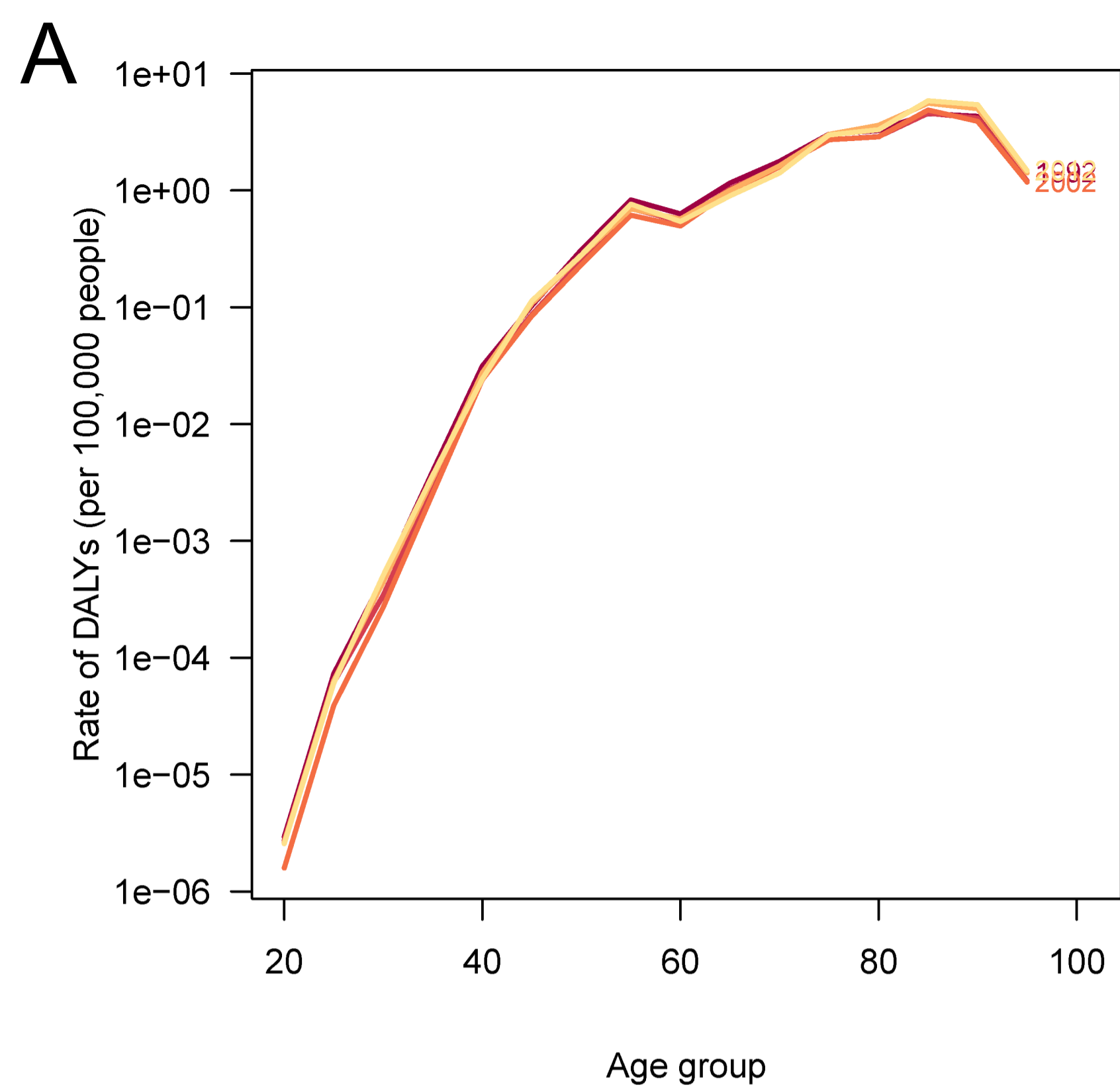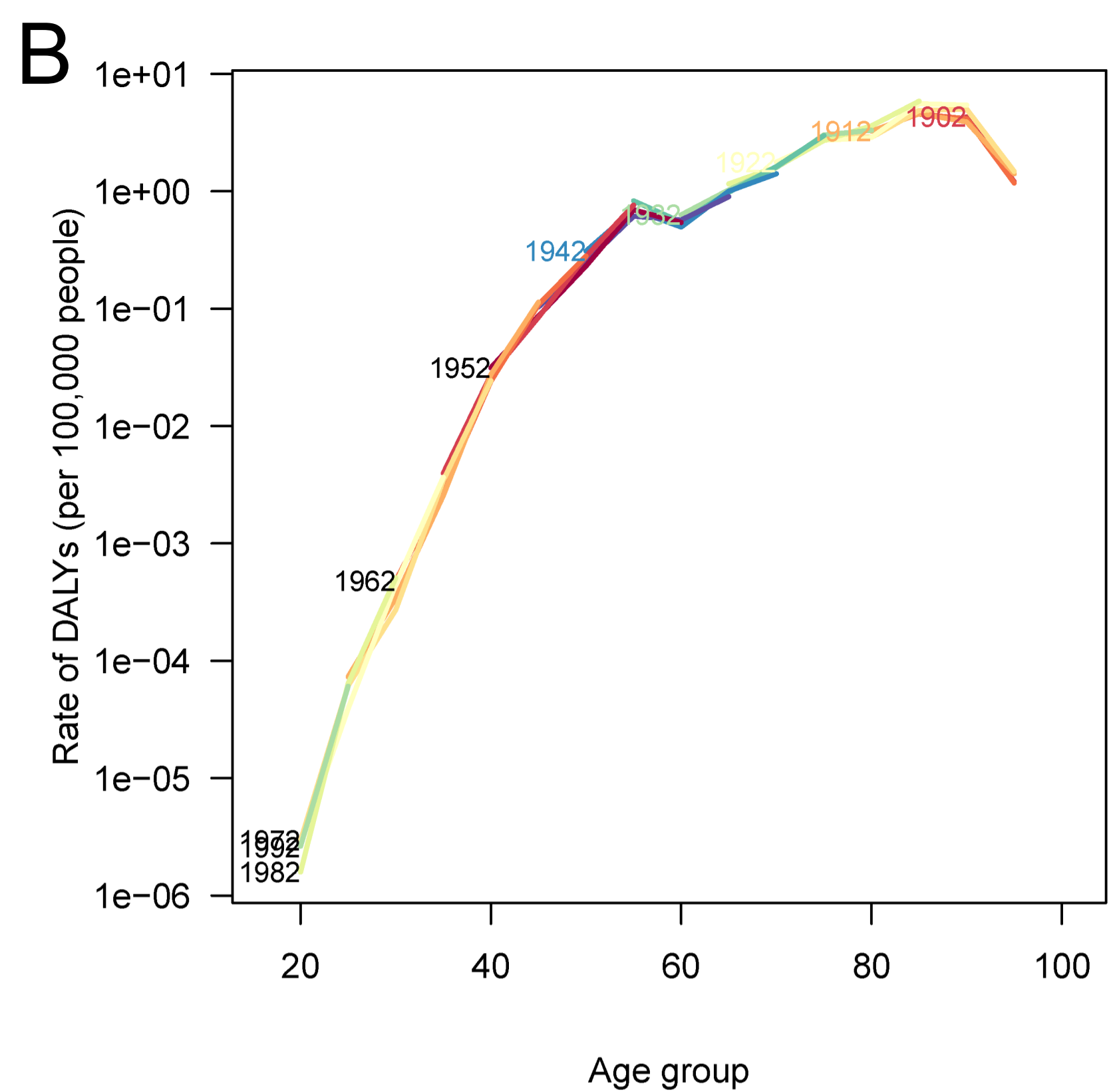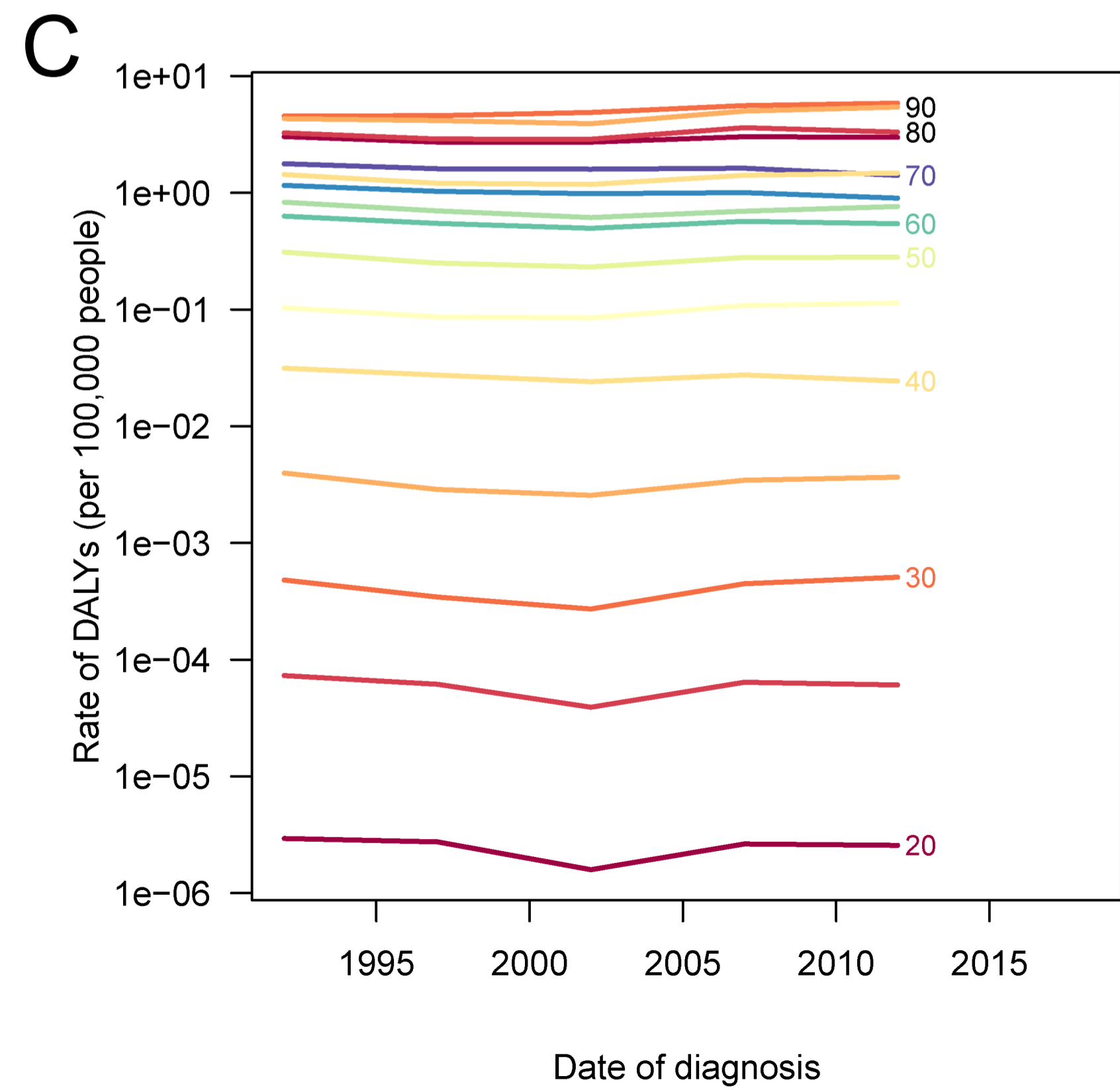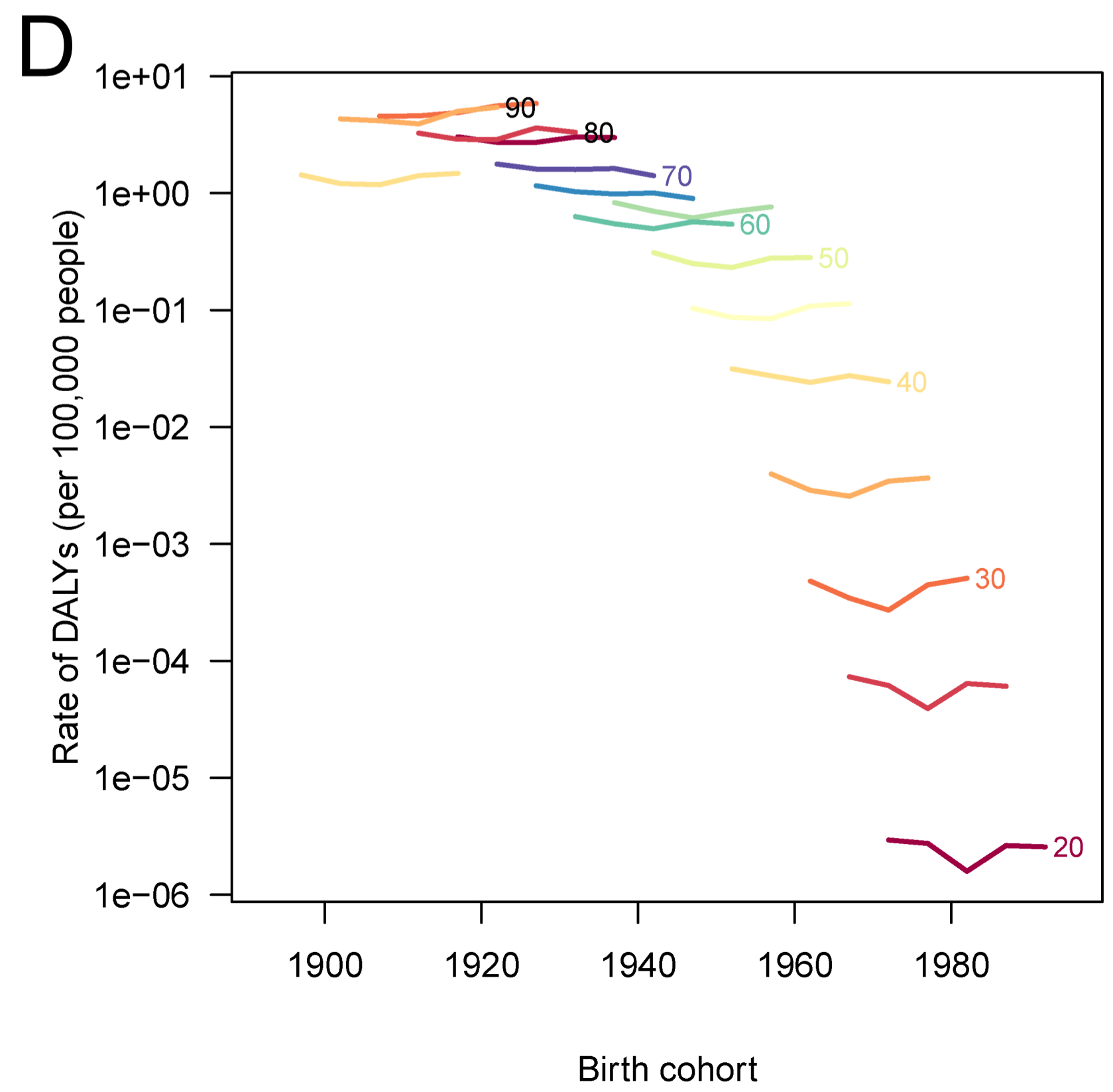

Supplement: S2 Fig — (A) Age-specific DALY rates according to time periods; each line connects the age-specific DALY rates for a 5-year period. (B) Age-specific DALY rates according to birth cohorts; each line connects the age-specific DALY rates for a 5-year birth cohort. (C) Period-specific DALY rates according to age groups; each line connects the period-specific DALY rates for a 5-year age group. (D) Birth cohort-specific DALY rates according to age groups; each line connects the birth cohort-specific DALY rates for a 5-year age group. Abbreviations: DALYs, disability-adjusted life years. (PDF) [file pone.0330878.s002.pdf]
