# Supplementary material for: Burden of laryngeal cancer attributable to occupational asbestos exposure in China: A comprehensive analysis from 1990 to 2021
Source: PLoS One. 2025 Aug 21;20(8):e0330878. doi: 10.1371/journal.pone.0330878 (PMC12370065; doi:10.1371/journal.pone.0330878)
Supplement: S1 Table — (DOCX) [file pone.0330878.s003.docx]

S1 Table. Trends in age-standardized mortality, DALY, YLD, and YLL rates (per 100,000 persons) among both sexes, males, and females from 1990 to 2021 for larynx cancer attributable to occupational asbestos exposure in China.

|  | Age-standardized mortality rate | | | Age-standardized DALY rate | | | Age-standardized YLD rate | | | Age-standardized YLL rate | | |
| --- | --- | --- | --- | --- | --- | --- | --- | --- | --- | --- | --- | --- |
| Gender | Period | APC (95% CI) | AAPC (95% CI) | Period | APC (95% CI) | AAPC (95% CI) | Period | APC (95% CI) | AAPC (95% CI) | Period | APC (95% CI) | AAPC (95% CI) |
| Both | 1990-1999 | -1.88 (-2.19 - -1.57) ^*^ | -0.99 (-1.29 - -0.70) ^*^ | 1990-1999 | -2.33 (-2.58 - -2.08) ^*^ | -1.17 (-1.41 - -0.92) ^*^ | 1990-1999 | -1.09 (-1.36 - -0.81) ^*^ | 0.68 (0.41 - 0.94) ^*^ | 1990-1999 | -2.36 (-2.61 - -2.11) ^*^ | -1.23 (-1.47 - -0.98) ^*^ |
|  | 1999-2006 | -0.21 (-0.76 - 0.35) |  | 1999-2006 | -0.44 (-0.89 - 0.01) |  | 1999-2004 | 1.23 (0.32 - 2.15) ^*^ |  | 1999-2006 | -0.51 (-0.96 - -0.06) ^*^ |  |
|  | 2006-2011 | 4.82 (3.71 - 5.93) ^*^ |  | 2006-2011 | 4.57 (3.67 - 5.47) ^*^ |  | 2004-2012 | 5.33 (4.92 - 5.75) ^*^ |  | 2006-2011 | 4.48 (3.58 - 5.40) ^*^ |  |
|  | 2011-2018 | -4.17 (-4.73 - -3.61) ^*^ |  | 2011-2018 | -4.03 (-4.48 - -3.57) ^*^ |  | 2012-2017 | -3.29 (-4.21 - -2.35) ^*^ |  | 2011-2018 | -4.10 (-4.55 - -3.64) ^*^ |  |
|  | 2018-2021 | -2.02 (-3.83 - -0.17) ^*^ |  | 2018-2021 | -1.84 (-3.33 - -0.32) ^*^ |  | 2017-2021 | -0.05 (-1.04 - 0.96) |  | 2018-2021 | -1.92 (-3.42 - -0.40) ^*^ |  |
| Female | 1990-1992 | -1.00 (-3.94 - 2.02) | -1.09 (-1.44 - -0.74) ^*^ | 1990-1992 | -1.46 (-4.17 - 1.32) | -1.26 (-1.57 - -0.95) ^*^ | 1990-1993 | -0.87 (-1.81 - 0.07) | 0.71 (0.52 - 0.90) ^*^ | 1990-1992 | -1.51 (-4.31 - 1.37) | -1.34 (-1.66 - -1.02) ^*^ |
|  | 1992-1998 | -3.88 (-4.49 - -3.26) ^*^ |  | 1992-1998 | -3.99 (-4.56 - -3.42) ^*^ |  | 1993-1998 | -3.05 (-3.60 - -2.50) ^*^ |  | 1992-1998 | -4.02 (-4.60 - -3.43) ^*^ |  |
|  | 1998-2007 | -0.00 (-0.31 - 0.31) |  | 1998-2007 | -0.06 (-0.35 - 0.23) |  | 1998-2006 | 2.12 (1.87 - 2.36) ^*^ |  | 1998-2007 | -0.15 (-0.45 - 0.16) |  |
|  | 2007-2011 | 5.12 (3.56 - 6.70) ^*^ |  | 2007-2011 | 4.19 (2.77 - 5.63) ^*^ |  | 2006-2011 | 6.70 (6.11 - 7.30) ^*^ |  | 2007-2011 | 4.05 (2.59 - 5.54) ^*^ |  |
|  | 2011-2015 | -5.28 (-6.71 - -3.82) ^*^ |  | 2011-2016 | -4.64 (-5.48 - -3.80) ^*^ |  | 2011-2016 | -2.75 (-3.32 - -2.18) ^*^ |  | 2011-2016 | -4.72 (-5.59 - -3.85) ^*^ |  |
|  | 2015-2021 | -1.09 (-1.64 - -0.54) ^*^ |  | 2016-2021 | -0.82 (-1.50 - -0.12) ^*^ |  | 2016-2021 | 0.97 (0.53 - 1.41) ^*^ |  | 2016-2021 | -0.90 (-1.61 - -0.19) ^*^ |  |
| Male | 1990-1999 | -1.69 (-2.07 - -1.32) ^*^ | -1.05 (-1.63 - -0.48) ^*^ | 1990-1999 | -2.18 (-2.46 - -1.90) ^*^ | -1.18 (-1.61 - -0.75) ^*^ | 1990-1999 | -1.00 (-1.28 - -0.71) ^*^ | 0.59 (0.31 - 0.86) ^*^ | 1990-1999 | -2.21 (-2.49 - -1.93) ^*^ | -1.24 (-1.67 - -0.80) ^*^ |
|  | 1999-2006 | 0.06 (-0.61 - 0.74) |  | 1999-2006 | -0.33 (-0.83 - 0.18) |  | 1999-2004 | 1.30 (0.35 - 2.25) ^*^ |  | 1999-2006 | -0.40 (-0.90 - 0.11) |  |
|  | 2006-2009 | 4.61 (0.54 - 8.85) ^*^ |  | 2006-2009 | 4.81 (1.72 - 7.99) ^*^ |  | 2004-2012 | 5.13 (4.71 - 5.54) ^*^ |  | 2006-2009 | 4.72 (1.61 - 7.92) ^*^ |  |
|  | 2009-2012 | 2.27 (-1.63 - 6.32) |  | 2009-2012 | 2.09 (-0.84 - 5.10) |  | 2012-2018 | -3.29 (-3.95 - -2.63) ^*^ |  | 2009-2012 | 2.03 (-0.92 - 5.07) |  |
|  | 2012-2018 | -5.16 (-6.01 - -4.30) ^*^ |  | 2012-2018 | -4.78 (-5.42 - -4.12) ^*^ |  | 2018-2021 | 0.25 (-1.45 - 1.99) |  | 2012-2018 | -4.84 (-5.50 - -4.19) ^*^ |  |
|  | 2018-2021 | -2.10 (-4.15 - 0.00) |  | 2018-2021 | -1.84 (-3.46 - -0.19) ^*^ |  |  |  |  | 2018-2021 | -1.92 (-3.55 - -0.27) ^*^ |  |

Abbreviations: AAPC, average annual percent change presented for full period; APC, annual percent change; CI, confidence interval. ^*^, *p* <0.05.
